# Development of Innovative Candied Chestnuts from Three Chestnut Cultivars Grown in Portugal

**DOI:** 10.3390/foods11070917

**Published:** 2022-03-23

**Authors:** Laure Foucher, Maria João Barroca, Yuliya Dulyanska, Paula M. R. Correia, Raquel P. F. Guiné

**Affiliations:** 1 Department of Food Industry, Agrarian School of Viseu, 3500-606 Viseu, Portugal; laurefoucher499@gmail.com (L.F.); ydulyanska@esav.ipv.pt (Y.D.); paulacorreia@esav.ipv.pt (P.M.R.C.); 2 University Institute of Technology, Angers University, 49035 Angers, France; 3 R&D Unit in Molecular Chemistry-Physics, Department of Chemistry, University of Coimbra, 3004-535 Coimbra, Portugal; mjbarroca@esac.pt; 4 Department of Food Technology, Polytechnic Institute of Coimbra, Coimbra College of Agriculture, Bencanta, 3045-601 Coimbra, Portugal; 5 CERNAS Research Centre, Department of Food Industry, Polytechnic Institute of Viseu, 3504-510 Viseu, Portugal

**Keywords:** chestnut products, new foods, candied chestnut, Portuguese cultivars

## Abstract

The main purpose of this work is the development of a value-added product (candied chestnuts) from Portuguese chestnut (*Castanea sativa*) cultivars (Martainha, Longal and Judia), as a way to minimize product loss and wastes. To accomplish this goal, the effects of rehydration, cooking, and syrup conditions on composition, textural properties, and colour parameters of candied chestnuts were investigated. The obtained results revealed that the optimal conditions to prepare candied chestnuts with a sweet taste, dark brown colour, with a crispy texture on the outside and smooth texture in the inner flesh were rehydration at 45 °C for a period of 5 h, cooking in a pressure pan for 15 min, and an immersion process with sucrose syrup for two days (syrup with 25% of sucrose on the first day and syrup of 50% of sucrose on the second day). During the process, the drying loss, hydration ratio, and cooking gain of the different cultivars were about 90%, 79%, and 130%, respectively. The total colour difference of candied chestnuts ranged from 24.18 (Longal) to 29.95 (Judia), the stickiness was moderately intense, and the adhesiveness was high for the three varieties. Longal candied chestnuts were the softest and Martainha candied chestnuts were the hardest, the most elastic, and cohesive. Moreover, the candied chestnuts presented a moisture content ranging from 52.70% and 54.23%, amounts of carbohydrates in the range of 88.58 to 91.87 g/100 g d.m, values of protein (6.55–9.51 g/100 g d.m.), values of ash (0.78–1.98 g/100 g d.m.), and fat (0.87–1.58 g/100 g d.m.). In conclusion, the chestnuts of Portuguese cultivars Martainha, Longal and Judia reveal a good potential to produce candied products with high added value.

## 1. Introduction

European chestnut (*Castanea* *sativa* Mill.) is one of the oldest and traditional edible fruits in Portugal and, in the past, was extensively used as a basic foodstuff until potatoes and other tubers became available [1]. Its use decreased over time, but in recent years consumers have shown an increased interest in nuts, particularly chestnuts, due to their nutritional qualities and potential health benefits. The chestnuts have high levels of healthy compounds (micronutrients, vitamins, antioxidants), low crude fat content, low saturated fatty acids, and high unsaturated fatty acids, namely linoleic and oleic acid [1,2,3,4,5]. Chestnuts are also rich in carbohydrates (mainly starch and sugars like sucrose) and have appreciable levels of fibre [1,6,7,8]. European sweet chestnut presents an interesting nutritional profile and can be also included in the diet of patients with diabetes (due to the high starch level as opposed to simple sugars) and celiac diseases since it is free from gluten [4,9]. However, chestnuts are seasonal fruits and are characterized by a limited shelf-life because of their high water activity and sugar content [6].

Depending on the chestnut variety, the nut can be used for fresh marketing, candying, drying and flour production [8]. Thus, chestnuts can be consumed in the fresh state (after being toasted and/or boiled) or as ingredients in a variety of processed foods (such as chestnut flour, mashed chestnuts and creams, yogurts and candied chestnuts) [5,8]. However, the consumption of nuts is still concentrated in a short period of time and connected to the traditional intake of nuts, toasted and/or cooked. Nonetheless, new forms of consumption are increasing, supported by the growing demand for specific dietary needs such as gluten free products and enriched products. Indeed, the combination of chestnut with other ingredients could be an interesting strategy to add value to fruit, minimize the losses in harvested fruits and provide a wider range of new products available to the consumer [10]. Among the high added value products based on chestnuts as raw material, “marrons glacées” are one of the most appreciated, namely in France, Italy, Switzerland, and Spain [5,11]. In the production of these sweet chestnuts the fruits are infiltrated or submerged in a sugar-rich solution and then covered with sugar syrups [5,11,12]. Other authors [10] combine freeze-drying Chinese chestnut (*Castanea mollissima*) and chocolate (dark and milk) coating, producing a product with an interesting profile and satisfactory bacteriological quality, extending the shelf life of chestnut for commercial application up to 12 months.

The project ValorCast-Valorization of chestnut and optimization of its commercialization aims to find answers to the problems of the chestnut chain starting on the production and following the different phases, like harvesting, processing, conditioning and storage, transportation and commercialization to reach the consumer. On average, the chestnut losses are around 15 to 30%, depending on the climatic conditions of the year and the management of the chestnut. This loss could represent around 10,000 tons of chestnuts (around 15 M €), out of a total of around 30,000 tons processed, which will have to be immediately diverted for scrap [13]. This high percentage, in addition to causing significant economic losses, raises serious problems by originating wastes or low-value products. Hence, it is required to find alternative ways to preserve the fruits, allowing them to be available all year-round while also contributing to the economic profit of farmers and processors. Following this need, the objective of this work was to develop a value-added product (candied chestnuts) from chestnuts (*Castanea sativa*) of Portuguese cultivars Martainha, Longal, and Judia, which are the three most relevant cultivars in the country. First, the conditions of rehydration, cooking, and syrup treatment to prepare the candied chestnuts with the expected taste, colour, and texture were optimized. Moreover, the composition, textural properties, and colour parameters of the candied chestnuts were evaluated.

## 2. Materials and Methods

### 2.1. Chestnut Samples

**Figure 1:**
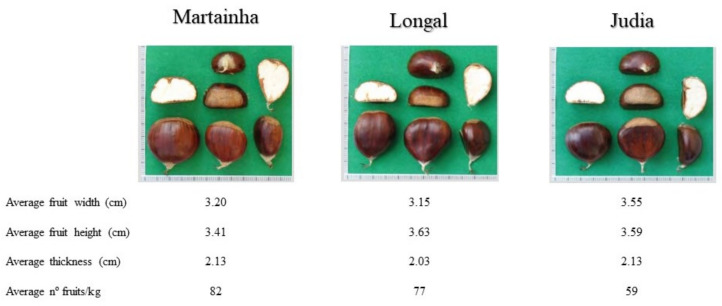
Chestnut cultivars native from Portugal used in the study [14].

The chestnut used in this study are from the variety *Castanea sativa*, cultivars Martainha, Longal, and Judia, which are some of the Portuguese cultivars with the highest expression in the country. Because of the importance of these varieties, they have been investigated to preserve their genetics and to promote these varieties for production and commercialization. Figure 1 shows the physical characteristics of varieties used and Table 1 presents their nutritional value.

**Figure 2:**
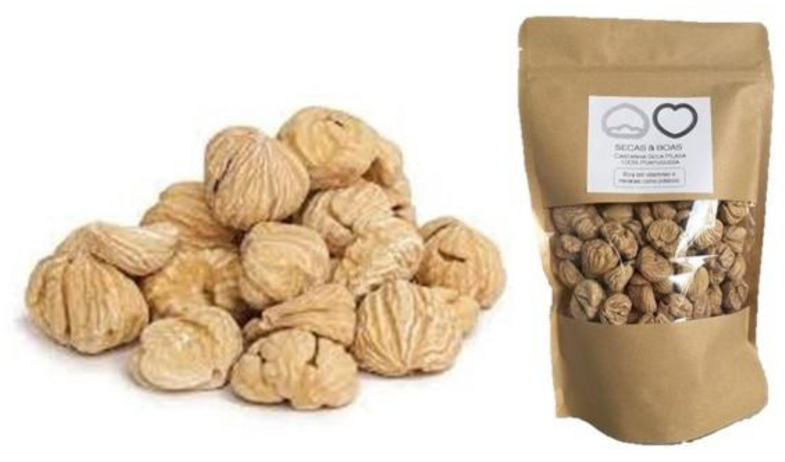
Dehydrate chestnut-“castanha pilada”.

The chestnuts used in the study were used in the dehydrated form known as “castanha pilada” in Portugal (Figure 2), which allows them to be preserved for use year-round. To obtain the dehydrated chestnuts, the shell and the inner skin were removed and then, drying occurred in a convective chamber (ventilated WTB-Binder) at 60 °C, with an air flow of 1 m/s for 48 h. The drying occurred in thin layer, with a load of 500 g per tray, arranged in a single layer of chestnuts over each of the four trays inside the dryer. 

### 2.2. Optimization of the Rehydration Process

The dehydrated chestnuts have a very low moisture content (below 2%, typically), which makes them safe for storage without any kind of degradation. However, this form of the product is too hard and not possible to chew or even process. For these reasons, their utilization year-round must be preceded by a rehydration process. The optimization process was made with chestnuts from only one variable (Martainha). Three tests were performed for the rehydration process at different temperatures of 25 °C, 45 °C, and 70 °C, and the evolution of moisture content was measured at different moments: after 1 h, 2 h, 3 h, 4 h, 5 h and finally 7 h.

Finally, the rehydration of the chestnuts from the three samples aimed at producing the candied chestnuts was carried out at optimal conditions chosen, based on the previous experiments, and which were soaked for a period of 5 h in water at 45 °C. The ratio of water to chestnut was 1000 mL to 100 g of dehydrated chestnuts.

### 2.3. Preparation of the Candied Chestnuts

The candied chestnuts to prepare are expected to have a sweet taste and dark brown colour, with a crispy texture on the outside and a smooth feeling of melting on the mouth for the inner flesh. For this, different recipes were pre-tested, and from one similar basic recipe changes were made step-by-step in order to encounter the best result. The pre-test was made again for only one cultivar of chestnut—Martainha.

- First essay: The rehydrated chestnuts were boiled during 15 min in a regular kitchen pan. In parallel, a syrup was prepared by boiling a mixture with 50% of sugar and 50% of water (m/m). Once the syrup is boiling the chestnuts were added and boiled for 2 min in the syrup. The chestnuts were left to stand in the syrup during the night (approximately 10 h). This procedure was repeated three consecutive days.
- Second essay: Because the normal cooking produced chestnuts which were too hard, the candied chestnuts obtained were too hard. Although possessing a pleasant sweet taste on the surface, the texture was not correct and much too hard. To compensate for this unacceptable texture, the cooking time was increased from 15 min to 30 min. Unfortunately, this did not change the texture of the products enough. Indeed, it was expected that with a longer cooking time the chestnuts would be saturated with water and therefore softer.
- Third essay: It was decided to change the soakings in the syrup. First day: put the chestnuts in a syrup with 25% sugar, boil the syrup with the chestnuts for 15 min in the morning and repeat the operation in the afternoon. Second day: repeat the procedure of the previous day but with a different concentration of sugar in the syrup: 50% sugar. These changes allowed the chestnuts to have a sweet taste on the surface and inside. However, the texture was not yet ideal.
- Fourth essay: In this attempt the soaking time in the syrup was increased from 15 min to 30 min for all steps. However, once again this change did not have a conclusive effect on texture.
- Fifth essay (FINAL): Analysing the previous results, it was concluded that with all attempts the chestnuts were always too hard, so it was hypothesized that cooking the chestnuts under pressure might give the desired texture. Hence, first the chestnuts were pressure cooked for 1 h, but the results showed they were much too soft and very brittle, losing their integrity. Because the chestnuts were unusable, the next test was conducted with a 30-min pressure cooking time. The chestnuts were a little more cohesive, but were still too fragile. Therefore, the optimal cooking time was established at 15 min in a pressure pan. At the end of this time duration, the chestnuts had an ideal texture, and had the appearance of properly cooked chestnuts.

**Figure 3:**
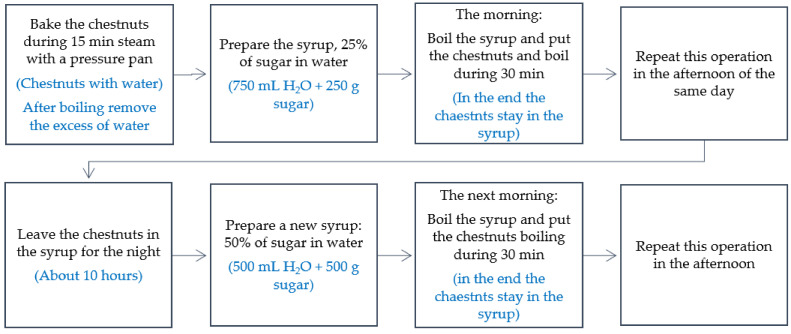
Final recipe for the production of candied chestnuts.

Figure 3 shows the final optimum procedure to obtain the candied chestnuts and which was used on the three types of chestnut studied.

**Figure 4:**
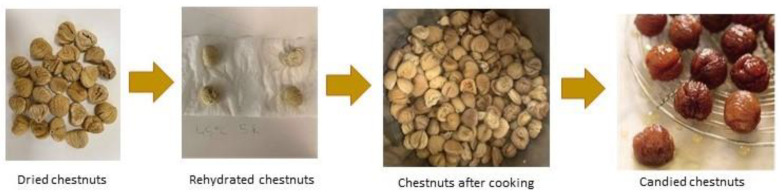
Martainha Chestnuts at different stages of processing.

Figure 4 shows the samples of chestnuts from cultivar Martainha at different stages.

### 2.4. Analyses of the Chemical Components

The values of moisture were evaluated through the method of drying until constant weight [15] and the average value and standard deviation were calculated from four separate measurements. The other chemical components, protein, fat, ash, fiber, and carbohydrates were all analysed following the Official Methods of Analysis of the AOAC International-Association of Official Analytical Collaboration [15]. In all cases and average value and corresponding standard deviation were obtained from four replicates.

### 2.5. Evaluation of Colour

For the evaluation of colour, measurements were made using a handheld device (Chroma Meter-CR-400, Konica Minolta, Japan). To evaluate the colour, we used the chromatic space of Cartesian Coordinates L* a* b* (CIELAB).

This method is based on psychological reality, that is, on brain perception and not on visual perception. It also uses the theory of opponents. This theory states that there are four fundamental colours (blue, yellow, red, green) that are opposed in pairs. The blue colour is therefore opposed to the yellow colour, the red colour is opposed to the green, and the white colour is opposed to the black.

Hence, the L* axis represents lightness and varies from 0 (corresponding to no lightness, i.e., absolute black), to 100, which is maximum lightness (i.e., absolute white). The other axes are represented by a* and b* and are at right angles to each other. The a* axis varies from green at one extremity (represented by −*a*) to red at the other (+*a*), whereas the b* axis varies from blue at one end (−*b*), to yellow (+*b*) at the other. Although in theory there are no extreme values of a* and b*, in practice they can frequently be numbered from −60 to +60. To perform the measurements care was taken to promote similar light conditions when measuring the colour of all samples, including the avoidance of direct sunlight and control of the incidence of artificial light. The calibration of the meter was done using a white tile, with illuminant D65 [16,17]. All measurements were made on ten replicates.

The total colour difference (Δ*E*) was calculated using Equation (1), which allows quantifying the overall colour difference between a sample and the reference, which is in natura chestnut sample:

(1) $$\Delta E=\sqrt{{\left(L^{*}-L_{0}^{*}\right)}^{2}+{\left(a^{*}-a_{0}^{*}\right)}^{2}+{\left(b^{*}-b_{0}^{*}\right)}^{2}}$$

where *L*_0_*, *a*_0_*, *b*_0_* are the colour coordinates for the reference sample [17,18,19].

A larger total colour difference corresponds to a greater colour change from the reference material, and one typical scale for evaluation of the colour difference is as follows [20]: Δ*E* values belonging to the interval [0.0–2.0] correspond to unrecognizable differences, Δ*E* values belonging to the interval [2.0–3.5] correspond to differences possible to recognize by an experienced observer and Δ*E* values higher than 3.5 correspond to clear differences in the colour of the samples under comparison.

### 2.6. Evaluation of Texture

To analyse the texture characteristics, a TA-XT2 texturometer (Stable Micro Systems, Godalming, UK) was used, and two types of tests were performed: a compression test (Texture Profile Analysis-TPA) and a cutting test. In all cases ten replicates were used from each sample.

#### 2.6.1. Cutting Test

**Figure 5:**
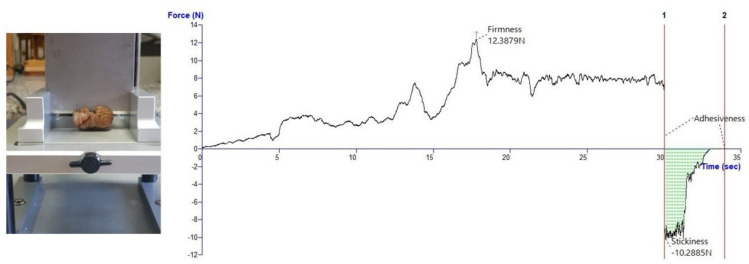
Texture analysis: curve force versus time for cutting test.

The cutting test was performed on the whole chestnut using a Blade Set with knife HDP/BSK (Warner-Bratzler). The test speed was 1.0 mm/s and the distance was 30 mm. Each analysis produced a curve of force versus distance, which allowed calculating three textural properties: firmness (force at highest peak), stickiness (force at lowest negative peak) and adhesiveness (area of the negative part of the curve) as shown in Figure 5.

#### 2.6.2. Compression Test (TPA—Texture Profile Analysis)

**Figure 6:**
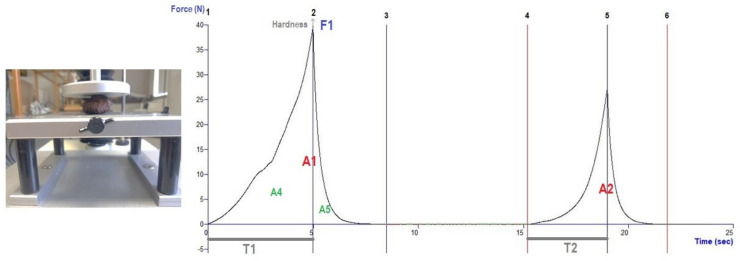
Texture analysis: curve force versus time for compression test (TPA).

The analysis of the texture profile consisted of two compression cycles using a cylindrical probe with a 75 mm base diameter (the probe being larger than the sample), spaced by an interval of 4 s between cycles. A 30 kg load cell was used and the test and post-test speeds were 1.0 mm/s. The compression distance was 5 mm and the trigger force was 0.1 N. The texture attributes: hardness, elasticity, resilience, cohesiveness and chewiness were calculated using the Equations (2)–(6), considering Figure 6 [21]

(2) $$\mathrm{Hardness}\;\left(N\right)=F1\;$$

(3) $$\mathrm{Springiness}\;\left(\%\right)=\frac{T2}{T1}\times 100\;$$

(4) $$\mathrm{Resilience}\;\left(\%\right)=\frac{A5}{A4}\times 100\;$$

(5) $$\mathrm{Cohesiveness}\;\left(\%\right)=\frac{A2}{A1}\times 100\;$$

(6) $$\mathrm{Chewiness}\;\left(N\right)=F1\times \frac{T2}{T1}\times \frac{A2}{A1}\;$$

### 2.7. Statistical Analysis

To test differences between mean values for multiple groups, an analysis of variance was undertaken (ANOVA), coupled with post-hoc test Tukey to identify differences when significant at the level of 5 % (*p* < 0.05). 

## 3. Results and Discussion

### 3.1. Rehydration

Table 2 presents the values of moisture for the three types of chestnut used, in the natural state, after drying and finally after the rehydration process. The values of moisture in natura are very similar for the three chestnut samples, and the drying substantially decreased the moisture content to values around 5–6%. These values of the moisture of “castanha pilada” are suitable to guarantee that no degradation reactions occur, either of microbial, chemical or enzymatic nature [22].

**Figure 7:**
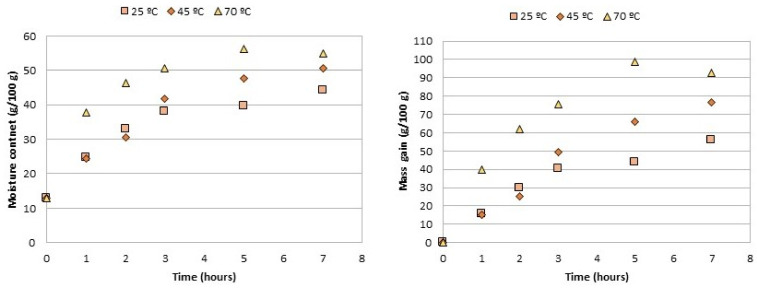
Rehydration procedure: variation of moisture content along time (**left**) and mass gain (**right**).

The dried form or the chestnuts, although very useful for storage and preservation of quality, is not proper for consumption or use in gastronomic preparations. Due to its extremely low moisture content (under 2%), this form of chestnut is incredibly hard, and not possible for human consumption or even for processing, except for milling and turning into a powder [6,14]. Therefore, either for direct consumption or for utilization in other gastronomic preparations, hydration must be carried out, and in this work the process was studied in order to optimize it. For the optimization of the rehydration process, tests were performed by dipping the chestnuts in water at three different temperatures (25 °C, 45 °C and 70 °C), and the moisture content was registered along rehydration (Figure 7). This allowed selecting the most appropriate temperature and time for the rehydration, that allows higher mass gain in a shorter time, while preserving the integrity of the fruits. These optimal conditions were, therefore, 5 h at a temperature of 45 °C. This choice was based on the experimental observations. It was observed that the fastest mass gain was at a temperature of 70 °C and that at 25 °C and 45 °C the curves are quite similar in the first 3 h. After 7 h of rehydration, at 70 °C the mass gain is much higher than the other two temperatures, and starts to decline thereof. The graph for moisture also shows that the higher the temperature, the faster the increase in the humidity level of the chestnuts. While for 70 °C the moisture content reached a maximum at 5 h and then decreased, for the other two temperatures the moisture content increased with temperature within the entire time range evaluated. From these results one could infer that highest mass gain would be obtained for a treatment at 70 °C for 5 h. Nevertheless, it was observed that, under these conditions, after rehydration, the samples did not hold, so they were not usable for being too fragile. Moreover, by rehydrating them at 70 °C, the physical-chemical properties of the chestnuts could be altered, such as for example by possible modifications in the starch structure or Maillard reactions of the sugars. At the temperature of 70 °C the gelatinization of starch can occur, thus modifying its crystalline or amorphous structure. The degree of starch gelatinization can reduce its digestibility in human gastrointestinal tract. Additionally, the degree of starch gelatinization has been associated with changes in food texture [23,24]. As for the Maillard reaction, which is a non-enzymatic browning reaction that takes place in presence of reducing sugars and amino acids, can take place at reduced temperatures, and is more intense as temperature raises [25]. Hence the chosen rehydration conditions to make the candied chestnuts involved leaving them in the bath for 5 h at 45 °C, which allows for obtaining a desirable moisture content while maintaining the integrity of the chestnuts. Then, this methodology was used to rehydrate the three samples and the moisture content was measured again after rehydration (Table 2). From the values of the rehydration ratio, it was possible to see that the chestnuts regained nearly 80% of the initial moisture, with the lowest value for the cultivar Judia (78.8%) and highest for Longal (79.6%). Still, the values are all very close. Once the chestnuts have been rehydrated, they can be cooked like fresh chestnuts. It was also observed that the cooking gain was around 130% for all samples.

### 3.2. Colour

The colour of the samples was measured as the first stage along the process, only for the cultivar Martainha, and the results are shown in Table 3. It is possible to observe that drying induces a high colour change, producing darker samples, as a result of browning processes, either of enzymatic or no-enzymatic nature, such as oxidation. A similar darkening for convective drying of food products has been reported: pomegranate arils [26], sarcocornia branches [27], eggplant [28], apple [29] or flour [30]. Also, the processing based on boiling with sugar syrup was responsible for quite an intense change in colour. This process involves the browning of the sugars, by caramelization processes and by Maillard reactions between the sugars and the amino acids from the proteins [31,32].

**Figure 8:**
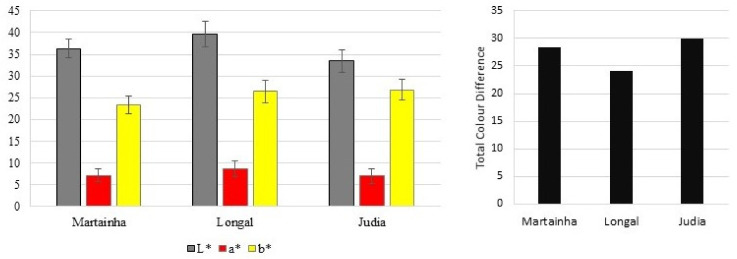
Colour coordinates for the three candied chestnuts (**left**) and total colour difference having the fresh fruits as reference (**right**).

At the second phase, the colour of all samples was evaluated, i.e., the three cultivars under study, as raw material and in final form of candied chestnuts made with fruits from cultivars Martainha, Longal and Judia. In this case, the evaluation of colour change was also performed to identify the intensity of the colour change as a whole (Figure 8). The colour parameters of the samples at the end of the caddying process is quite similar, with lightness varying from 33.45 ± 2.64 for Judia (the darkest) and 39.65 ± 3.04 for Longal (the clearest), redness varying from 7.01 ± 1.64 for Judia and 8.62 ± 1.87 for Longal (the sample with more intense red colouration), and yellowness varying from 23.44 ± 2.05 for Martainha and 26.85 ± 2.37 for Judia (the sample with more intense yellow). Regarding the total colour difference, measured in relation with the fresh samples, i.e., prior to the processing operations, the values are very high, corresponding in all cases to clear differences easily identified by non-experienced observers [20]. The values of Δ*E* in our study vary in the range 24.18–29.95, for a convective drying performed for 48 h at 60 °C. Barroca et al. [27] observed a temperature for the drying of sarcocornia also at 60 °C, but a lower drying time of only 7 h and a value of Δ*E* equal to 17.84. In our case the Δ*E* is higher because our samples remained in contact with the hot air for a longer period and that induced more intense changes in colour. Additionally, they were submitted to the candying process of boiling with the sugars, which contributed to the intensification of the brownish colouration.

### 3.3. Texture

In the stage of process optimization, the texture of the products (for Martainha cultivar) was measured at different phases, and for different cooking options. These values helped in choosing the best operation conditions to obtain the candied chestnuts. The textural parameters for process optimization are shown in Table 4. Properties such as hardness or firmness increased with drying and decreased after hydration, but were most affected by the cooking process. Still, while boiling for 15 min was not enough to soften the texture, and the increase to 30 min practically did not change this fact, the use of the pressure cooking substantially decreased hardness and firmness, so that the samples were much too soft to be able to be processed further. For this reason, the pressure cooking for only 15 min allowed the best textural properties. It was further observed that the candied chestnuts had very high adhesiveness and stickiness, as a result of the sugars present in the surface of the product. These sugars undergo caramelization reactions, which increase stickiness and adhesiveness (Wang and Hartel 2021), besides other textural or rheological changes. Regarding chewiness, it greatly decreased with processing from 35.43 ± 4.72 N in the raw fruits to only 2.09 ± 0.56 N in the candied chestnuts. Regarding resilience, it remained pretty unchanged, varying from 21.24 ± 0.54% for the hydrated sample to 32.12 ± 1.25% for the raw chestnuts. According to Moreira et al. [9] the rehydration of dried chestnuts results in modifications both in the colour and textural properties of the chestnuts.

**Figure 9:**
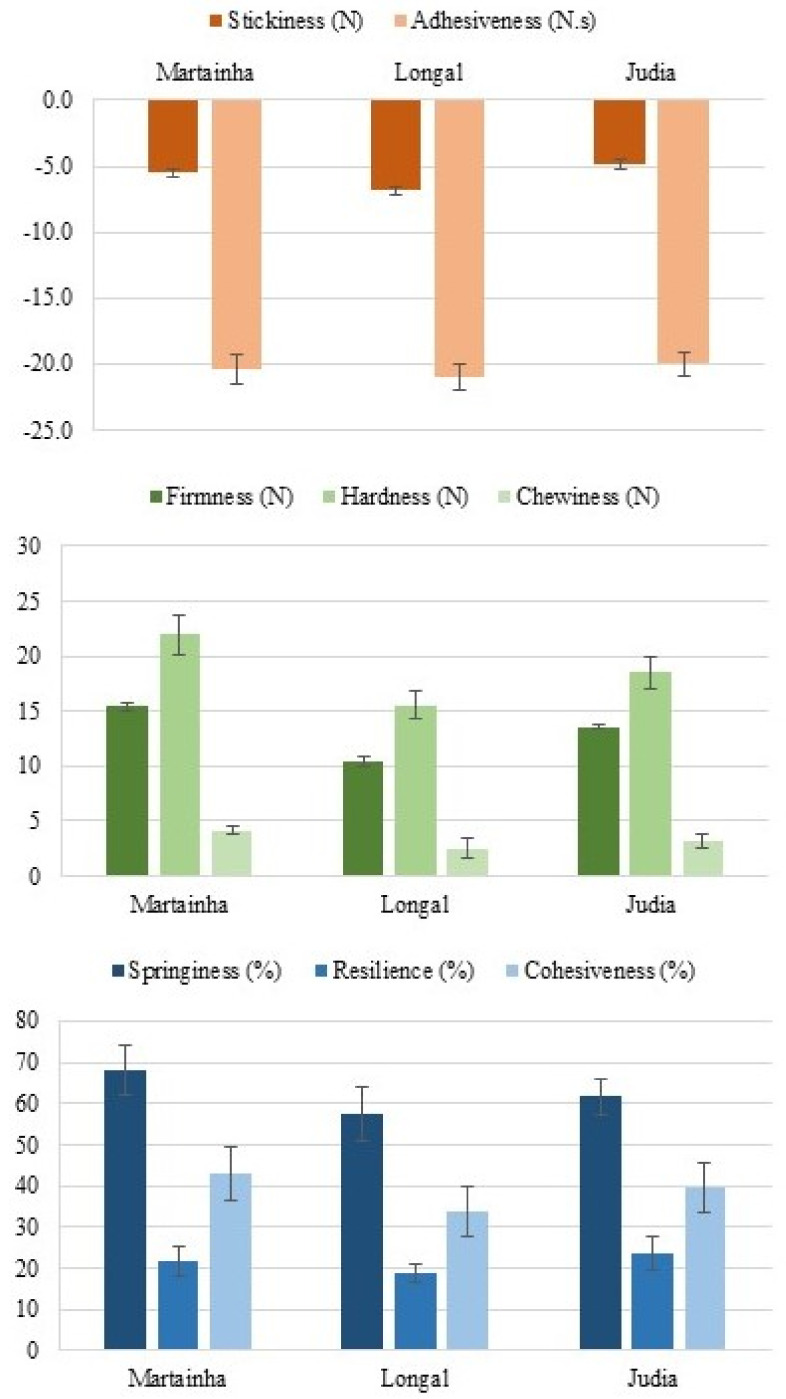
Textural parameters of the candied chestnuts.

When the four types of chestnuts were candied, the textural properties were also measured and the results are presented in Figure 9. Stickiness was moderately intense for the three samples (values in the range −6.87 ± 0.30 to −4.87 ± 0.39 N) and adhesiveness was very high (absolute values varying from 20.01 ± 0.85 to 21.04 ± 0.96 N.s). The results further showed that the hardest sample was Martainha (21.94 ± 1.78 N) and the softest was Longal (15.54 ± 1.25 N). Finally, the Martainha candied samples were those with higher springiness (67.91 ± 5.98%), being more elastic, and also more cohesive (42.81 ± 6.54%). The presence of starch and amylose in the chestnuts influences their textural properties during thermal processing [33,34]. In the present case, the samples had a high content of starch globally, and some minor differences between cultivars (Table 1).

### 3.4. Chemical Composition and Nutritional Value

The chemical composition and nutritional value of the candied chestnuts obtained with the three cultivars of chestnut are presented in Table 5. The results showed that the proximate composition of the three samples is very similar and so are the energetic values (varying from 184 to 190 kcal). Although there are no apparent differences in the contents of moisture, carbohydrates, ash, fat or protein, for the candied chestnuts obtained with Martinha, Longal and Judia fruits, one can easily detect some differences in relation to the fresh samples (data in Table 1). These differences can be due to the processing operations or also due to the methodologies used in the analyses. The candied chestnuts presented amounts of carbohydrates in the range 88.58 to 91.87 g/100 g d.m., which are higher than those reported by Costa et al. [14] for the raw chestnuts of the same cultivars (Table 1). Also, the values of protein for the candied chestnuts (6.55–9.51 g/100 g d.m.) were higher than those reported for the raw fruits (4.9–5.6 g/100 g d.m.) [14]. On the other hand, the candied chestnuts possessed lower values of ash (0.78–1.98 g/100 g d.m.) and fat (0.87–1.58 g/100 g d.m.), as compared with the fruits in natura of the same cultivars (ranges 1.7–1.8 and 2.4–3.4 g/100 g d.m., respectively, for ash and fat).

## 4. Conclusions

The results obtained in this work allowed for the establishing of the best conditions to use the chestnuts from the varieties studied, for the production of candied chestnuts. Hence, the optimal conditions to prepare candied chestnuts with a sweet taste, dark brown colour and with a crispy texture on the outside and smooth in the inner flesh, include soaking the fruit in water for a period of 5 h at 45 °C, cooked in a pressure pan for 15 min, and immersed in sucrose syrup for two days. During the process, the drying loss, hydration ratio, and cooking gain of the different cultivars were about 90%, 79% and 130%, thus allowing them to gain enough water to be of use in the processing.

The candied chestnuts, of the three varieties, presented a total colour difference ranging from 24.18 (Longal) to 29.95 (Judia), moderate stickiness, and intense adhesiveness, due to the presence of the sugar. Martainha candied chestnut was hard, elastic, and cohesive, while Longal candied chestnut was soft. These properties make the different varieties appropriate for use to obtain final products with different organoleptic characteristics, and thus maybe please different consumer districts. Candied chestnuts obtained with Martainha, Longal, and Judia fruits presented a similar nutritional profile.

To conclude, the chestnuts of Portuguese cultivars Martainha, Longal, and Judia have good potential to produce candied products with high added value.

## Figures and Tables

**Table 1:** Nutritional characterization of the regional cultivars of chestnut [14].

Components	Martainha	Longal	Judia
Macro components (g/100 g d.m.) *			
Total sugars	37.3 ± 1.1	38.8 ± 1.6	31.5 ± 1.8
Ash	1.8 ± 0.1	1.7 ± 0.0	1.7 ± 0.1
Fat	2.4 ± 0.2	2.7 ± 0.2	3.4 ± 0.1
Protein	4.9 ± 0.6	5.3 ± 0.4	5.6 ± 0.5
Fibre	3.9 ± 0.2	3.4 ± 0.1	3.3 ± 0.1
Starch	39.3 ± 2.3	38.5 ± 1.1	39.5 ± 1.7
Vitamins (mg/100 g d.m.) *			
Thiamine (B_1_)	1.19 ± 0.03	0.75 ± 0.07	1.13 ± 0.09
Riboflavin (B_2_)	0.26 ± 0.02	0.45 ± 0.01	0.37 ± 0.02
Niacin (B_3_)	4.18 ± 0.09	5.6 ± 0.03	5.40 ± 0.04
Ascorbic acid (Vit. C)	121.0 ± 0.0	104.0 ± 0.1	111.0 ± 0.4

* Values expressed as mean value ± standard deviation per 100 g of dry matter.

**Table 2:** Moisture content of the regional cultivars of chestnut used.

Moisture Content (g/100 g)	Martainha	Longal	Judia
In natura (before convective drying) ^1^	60.03 ± 0.73 a	59.71 ± 1.05 a	60.96 ± 0.12 a
After drying ^1^	6.00 ± 0.62 ab	6.44 ± 0.98 a	5.36 ± 0.32 b
After rehydration ^1^	47.63 ± 1.98 a	47.52 ± 2.04 a	48.01 ± 2.10 a
After cooking ^1^	62.10 ± 2.24 a	61.85 ± 2.17 a	62.45 ± 1.87 a
After syrup treatment ^1^	54.11 ± 1.67 a	55.61 ± 1.98 a	54.57 ± 2.04 a
Drying loss (%) ^2^	90.0	89.2	91.2
Hydration ratio (%) ^3^	79.3	79.6	78.8
Cooking gain (%) ^4^	130.4	130.2	130.1

^1^ Values in the same line with the same letter are not significantly different (ANOVA with test Tukey, *p* < 0.05). ^2^ Drying loss = (initial moisture-moisture after drying)/initial moisture × 100 %. ^3^ Hydration ratio = moisture after rehydration/initial moisture × 100 %. ^4^ Cooking gain = moisture after cooking/moisture after rehydration × 100 %.

**Table 3:** Colour coordinates along the process and for different cooking options for cultivar Martainha.

Textural Parameters	L*	a*	b*	Δ*E* ^1^
In natura	62.31 ± 4.22 a	7.35 ± 1.61 b	34.83 ± 2.50 a	-
Dried	36.71 ± 5.16 e	14.98 ± 3.74 a	26.48 ± 5.44 c	27.99
Hydrated	56.98 ± 3.50 b	4.03 ± 1.25 c	26.04 ± 3.51 c	10.46
Boiled 15 min	57.55 ± 4.82 b	4.21 ± 1.42 c	27.12 ± 3.41 bc	9.59
Boiled 30 min	58.14 ± 5.52 b	4.42 ± 0.89 c	29.14 ± 2.99 b	7.64
Pressure cooked 1 h	54.71 ± 4.66 c	4.10 ± 2.06 c	26.88 ± 4.18 b	11.47
Pressure cooked 30 min	55.43 ± 6.73 bc	4.13 ± 1.65 c	27.04 ± 3.74 b	10.89
Pressure cooked 15 min	54.16 ± 6.48 c	3.95 ± 1.63 c	25.88 ± 3.81 c	12.58
Candied	42.79 ± 4.95 b	8.07 ± 2.02 b	24.79 ± 4.26 d	21.96

Values in the same column with the same letter are not significantly different (ANOVA with test Tukey, *p* < 0.05). ^1^ Δ*E* was calculated with the fresh sample (in natura) as reference.

**Table 4:** Textural parameters along the process and for different cooking options.

Textural Parameters	In Natura	Dried	Hydrated	Cooked					Candied
				Boiled 15 min	Boiled 30 min	Pressure 1 h	Pressure 30 min	Pressure 15 mim	
Cut test									
Firmness (N) ^1^	152.04 ± 12.04 b	185.64 ± 10.21 a	116.91 ± 7.86 c	64.08 ± 7.23 d	59.16 ± 5.42 d	3.61 ± 1.40 h	6.07 ± 0.36 g	15.16 ± 1.14 e	10.41 ± 3.09 f
Stickiness (N) ^1^	−0.51 ± 0.30 a	−0.23 ± 0.12 a	−1.74 ± 0.41 b	−1.75 ± 0.24 b	−2.03 ± 0.23 c	−2.63 ± 0.11 c	−2.40 ± 0.14 c	−2.28 ± 0.12 c	−4.99 ± 0.18 d
Adhesiveness (N) ^1^	−2.07 ± 0.81 a	−1.77 ± 0.49 a	−6.66 ± 0.56 b	−14.32 ± 1.16 e	−14.77 ± 0.93 e	−15.20 ± 1.33 e	−12.8 ± 0.85 d	−9.48 ± 2.81 c	−23.59 ± 0.51 f
Compression test									
Hardness (N) ^1^	375.42 ± 24.12 b	451.57 ± 19.24 a	128.39 ± 9.97 c	80.54 ± 6.41 d	62.04 ± 4.85 e	12.32 ± 2.74 h	12.75 ± 1.84 h	19.25 ± 3.99 f	15.81 ± 3.94 g
Springiness (%) ^1^	45.78 ± 4.85 c	44.26 ± 6.71 c	71.29 ± 5.41 a	55.46 ± 8.12 b	56.41 ± 4.52 b	57.21 ± 2.84 b	55.41 ± 6.12 b	54.71 ± 9.14 b	48.26 ± 4.27 c
Resilience (%) ^1^	32.12 ± 1.25 a	29.58 ± 1.46 b	21.24 ± 0.54 d	25.69 ± 4.12 c	23.54 ± 3.17 c	26.51 ± 2.74 c	26.98 ± 5.12 c	25.89 ± 3.85 c	27.25 ± 4.58 c
Cohesiveness (%) ^1^	21.54 ± 4.95 c	23.71 ± 4.78 c	58.43 ± 7.52 a	25.41 ± 5.71 c	24.78 ± 6.11 c	26.43 ± 5.55 c	27.23 ± 2.48 b	25.95 ± 5.48 c	29.41 ± 5.88 b
Chewiness (N) ^1^	35.43 ± 4.72 b	47.63 ± 5.66 a	33.36 ± 4.62 b	11.05 ± 3.41 c	8.33 ± 1.77 d	1.78 ± 0.09 e	1.92 ± 0.44 e	1.25 ± 0.87 e	2.09 ± 0.56 e

^1^ Values in the same line with the same letter are not significantly different (ANOVA with test Tukey, *p* < 0.05).

**Table 5:** Nutritional composition of the candied chestnuts.

Composition	Candied Martainha ^1^	Candied Longal ^1^	Candied Judia ^1^
Moisture ^2^	52.70 ± 0.14 c	53.17 ± 0.30 b	54.23 ± 0.57 a
Carbohydrates ^2^	41.90 ± 1.27 ab (88.58)	41.65 ± 0.49 b (88.94)	42.05 ± 0.78 a (91.87)
Ash ^2^	0.84 ± 0.14 b (1.78)	0.93 ± 0.02 a (1.98)	0.36 ± 0.15 c (0.78)
Fat ^2^	0.45 ± 0.07 b (0.95)	0.74 ± 0.16 a (1.58)	0.40 ± 0.00 c (0.87)
Protein ^2^	4.50 ± 0.28 a (9.51)	3.85 ± 0.49 b (8.22)	3.00 ± 1.41 b (6.55)
Energy (kcal/100 g)	190	189	184
Energy (J/100 g)	794	790	769

^1^ Values expressed as mean value ± standard deviation in g/100 g of sample (mean expressed in dry matter as g/100 g d.m.). ^2^ Values in the same line with the same letter are not significantly different (ANOVA with test Tukey, *p* < 0.05).

## Data Availability

The data are available from the corresponding author upon reasonable request.
